# PARP12-mediated ADP-ribosylation contributes to breast cancer cell fate by regulating AKT activation and DNA-damage response

**DOI:** 10.1007/s00018-025-05586-z

**Published:** 2025-01-23

**Authors:** Anupama Pavithran, Maria Matarese, Barbara Morone, Angela Filograna, Matteo Lo Monte, Nina Alayne Dathan, Daniela Corda, Giovanna Grimaldi

**Affiliations:** 1 Institute of Endotypes in Oncology, Metabolism, and Immunology, National Research Council, Via Pietro Castellino 111, Naples, Italy; 2 OU Health Stephenson Cancer Center, Oklahoma, USA

**Keywords:** PARP12, Breast cancer, AKT, p53, Apoptosis, DNA-damage

## Abstract

Breast cancer represents the primary cause of death of women under 65 in developed countries, due to the acquisition of multiple drug resistance mechanisms. The PI3K/AKT pathway is one of the major regulating mechanisms altered during the development of endocrine resistance and inhibition of steps in this signalling pathway are adopted as a key strategy to overcome this issue. ADP-ribosylation is a post-translational modification catalysed by PARP enzymes that regulates essential cellular processes, often altered in diseases. PARP12, a member of this family, has been associated with the onset of drug resistance in oestrogen receptor-positive breast cancers, making this enzyme a promising drug target. The molecular basis underlying its involvement in the acquisition of resistance are unknown to date. Here, we demonstrate that PARP12-mediated mono-ADP-ribosylation of AKT is required for AKT activation whilst the absence of PARP12 leads to apoptosis induction in a subset of oestrogen receptor-positive breast cancer cells. Our data show that transcriptional inhibition of PARP12 correlates with an increased DNA-damage induction, mirrored by augmented p53 nuclear localisation and enhanced p53-AKT interaction. Under these conditions, AKT is functionally incompetent towards its downstream targets FOXO, hence favouring cell death. This is achieved by increasing protein levels of the FOXO1 transcription factor, that in turn activates the apoptotic cascade. Overall, we show a novel regulation step of AKT activation and apoptosis relying on PARP12-mediated mono-ADP-ribosylation and propose PARP12 as a potential pharmacological target to be exploited as an innovative therapeutical strategy to overcome endocrine resistance.

**Supplementary Information:**

The online version contains supplementary material available at 10.1007/s00018-025-05586-z.

## Introduction

Breast cancer is the leading cause of death of women under 65 in the Western World. Breast cancers are highly heterogenous and the molecular signature dictates the choice of therapeutic interventions. Most breast cancers harbour the oestrogen receptor (ER) and rely on ER-driven signalling for proliferation and survival [1, 2]. Hormone-based therapy is therefore the standard of care for ER positive (ER +) breast cancers; despite its high success rate, still a significant percentage of patients develop drug-resistance [3]. The constitutively active PI3K/AKT pathway is one of the main deregulated processes associated with the onset of drug-resistance and pharmacological interventions targeting this pathway are currently used in combinatorial therapies [4].

Here, we have investigated a novel regulating step of AKT activation, mediated by the mono-ADP-ribosyltransferase PARP12. PARPs constitute a family of enzymes catalysing ADP-ribosylation, a covalent chemical modification highly conserved throughout evolution [5, 6]. The reaction consists of the transfer of ADP-ribose from nicotinamide adenine dinucleotide (NAD +) onto the substrate with subsequent release of nicotinamide [6, 7]. The modification was initially identified as a post-translational protein modification, and later also found to occur on nucleic acids [8, 9]. Members of the PARP family can either catalyse transfer of a single unit of ADP-ribose (mono-ADP-ribosylation, hereafter referred as MARylation) or transfer multiple units of ADP-ribose, to form long and branched polymers of ADP-ribose (poly-ADP-ribosylation, hereafter referred as PARylation). PARylation is catalysed by PARP1, -2 and tankyrase enzymes, whilst MARylation is catalysed by PARP 3,4,6–12 and PARP 14–16 [6]. PARPs regulate key cellular functions, most of which are relevant to cancer, such as regulation of chromatin structure, DNA damage repair and cellular stresses, as well as regulation of cell cycle progression [6, 10]. The mono-ADP-ribosyltransferase PARP12 is encoded by an interferon stimulated gene and has mainly been studied for its role as an anti-viral factor [5, 11–16]. It also exerts key functions in intracellular trafficking, by regulating exocytic and endocytic processes [5, 17]. Along with these roles, PARP12 has been identified as one of the main factors involved in the acquisition of breast-cancer resistance to chemotherapy, evidenced through a delayed cancer cell colony regrowth upon transcriptional inhibition of PARP12 [18]. The present study investigates PARP12 function in breast cancer cells, to clarify the molecular details of PARP12’s role in enhanced cell survival. Our data identify the mono-ADP-ribosylation catalysed by PARP12 as a novel regulatory step in AKT activation. Transcriptional inhibition of PARP12 causes a reduction in AKT phosphorylation levels on both threonine 308 and serine 473 residues. As a result of inactive AKT, protein levels of FOXO transcription factors increase, leading to the up-regulation of pro-apoptotic genes and induction of DNA-damage, with consequent increased cell death.

## Methods

### Antibodies and reagents

All antibodies used were rabbit polyclonal unless specified. Commercially available antibodies: GAPDH (Abcam), PARP12 goat polyclonal (Abcam) (IF: 1:200), PARP12 (Sigma Prestige Antibody), 6X His-Tag mouse monoclonal (Invitrogen), GST mouse monoclonal (Abcam), HA mouse monoclonal (New England Biolabs), p53 mouse monoclonal (DO-1) (Santa Cruz; IP 2 µg/1 mg lysate)**,** anti-Golgin-97(1:2,000) (kindly provided by Dr. Antonella De Matteis-TIGEM-Pozzuoli, Naples), MAVS (IF: 1:400), HA- Rabbit monoclonal (C29F4)**,** total AKT [IP: 1:100 (v/v); IF: 1:100)], total AKT mouse monoclonal (clone 40D4; IF:1:200), γH2A.X (phospho-S139) mouse monoclonal (N1-431, Abcam, IF: 1:500), Poly-Mono-ADP-Ribose rabbit monoclonal (E6F6A), PARP1, Bim, pAKT (T308), pAKT (S473), pFOXO1 (S256), total FOXO1, p53 mouse monoclonal (IF: 1:200), β-actin mouse monoclonal, normal IgG mouse. All the above antibodies were purchased from Cell Signalling Technology unless otherwise specified. For WB, all primary antibodies were diluted 1:1000, except for GAPDH, 6X-His Tag, GST, β-Actin (1:10,000), p53 (DO-1) (1:500) and HA (1:4,000). HRP-conjugated secondary antibodies were purchased from Calbiochem and used 1:5,000 final dilution; Alexa Flour-488, -568 and -647 conjugated secondary antibodies for immunofluorescence were purchased from Molecular Probe and used 1:400 final dilution. AKT inhibitor MK-2206 was purchased from Selleckem. Olaparib, Protein A-Sepharose CL-4B (GE17-0780–01, Cytiva). PJ-34 Hydrochloride Hydrate, PhosoStop (phosphatase inhibitor cocktail), cOmplete (TM)#ULTRA Tablets (protease inhibitor cocktail), Glutathione Sepharose 4B, Immobilon Crescendo Western HRP Substrate, mPAGE™ Bis–Tris Precast Gel 8%,10%, 4–12% were purchased from Merck Millipore. IMMOBILON-P, Protran Pure Nitrocellulose Membrane [45 µm] was from Revvity.

### Cell cultures and transfection

MCF7 cell line was purchased from European Collection of Authenticated Cell Cultures (ECACC), MCF10A, MDA-MB-231, PC3 cell lines were purchased from the American Type Culture Collection (ATCC). ZR-75-1, MDA-MB-468 and HCT-116 cell lines were kindly provided by dr. Nunzio Antonio Cacciola (Federico II University of Naples, Italy), HepG2 cell lines were originally obtained from the laboratory of Dr. Alberto Luini (IEOMI, National Research Council, Naples, Italy). MCF7, MDA-MB-231 and HepG2 cells were cultured in DMEM medium. The cell lines ZR-75-1, MDA-MB-468, HCT-116 cells were grown in RPMI medium, while PC3 cells were cultured in DMEM-F12 medium. All the media listed above were supplemented with 10% (v/v) FBS, 2 mM L-glutamine. MCF10A cells were cultured in DMEM-F12 media supplemented with 5% horse serum, 20 ng/ml epidermal growth factor, 500 ng/ml hydrocortisone, 100 ng/ml cholera toxin, 10 μg/ml insulin and 2 mM L-glutamine. All the above supplements and media were purchased from GibcoTM. All cell lines were cultured in a 37 °C and 5% CO_2_ incubator. The AKT wild-type or its mutant constructs were transiently transfected using Lipofectamine LTX Reagent with PLUS Reagent (Invitrogen) in Opti-MEM^TM^ medium (GibcoTM). The cDNA for the reaction was used at a concentration of 6 μg for WT and M4 AKT, 7.2 μg for M1, M2, M5, 5.5 µg phospho-MUT AKT. The cells were then incubated for 24 h, in the same growth conditions. For RNA interference, commercially available siRNA oligos targeting hPARP12 (Dharmacon) were used at a concentration of 50 nM in Opti-MEM™ medium and transfected with Lipofectamine™ RNAiMAX Transfection Reagent (Invitrogen) for all cell lines, except HepG2 and HCT116, where Oligofectamine was used as transfection reagent. To transiently knock down human p53, cells were transfected with a p53 siRNA (5′-GGAAAUUUGCGUGUGGAGU-3′) as previously described [19]. MCF10A cell line was transfected using 100 nM of hPARP12 siRNA oligos. The cells were then incubated for 24 h, 48 h, 72 h in normal growth conditions. The efficiency of the interference was assessed by western blot analysis. For PARP12 knock-down experiments, 2 × 10^6^ MCF7 cells were plated in 100 mm of diameter cell culture plates and PARP12 siRNA transfection performed for 48 h before performing immunoprecipitation assay of endogenous p53. In all the other cases, PARP12 knock-down was performed when cell confluence was in the range of 50% to 70%, independently of the cell line.

### Flow cytometry

MCF7 cells were seeded into 6 well plates (3 × 10^5^ cells). After overnight (ON) incubation at 37 °C, the cells were transfected or not with PARP12 siRNAs for 24 h, 48 h and 72 h. Trypsinised cells were pelleted, washed in ice-cold PBS, resuspended in ice-cold ethanol (while vortexing) and then incubated overnight at −20 °C. Then, the samples were centrifuged at 500×*g* for 5 min, the ethanol was removed, and the cells were washed in ice-cold PBS and incubated with 50 μg/ml propidium iodide for 30 min in the presence of 200 μg/ml RNAse A. The cells were then analysed using the Becton Dickinson (BD) FACS-CantoA instrument. Data are means ± SD of three independent experiments.

### Apoptotic assay

Apoptosis-mediated cell death of MCF7 cells was examined at 48 h and 72 h following PARP12 siRNAs transfection by double staining with the Annexin V-FITC kit (Miltenyi Biotec GmbH), according to the manufacturers’ instructions. Data are means ± SD of three independent experiments.

#### Real-time apoptosis acquisition

Incucyte SX1 Live-Cell Imaging system (Sartorius) was used for kinetic monitoring of apoptotic activity of MCF7 cells. Approximately 2 × 10^4^ MCF7 cells were plated onto a 48-well plate and transiently transfected with PARP12 siRNAs (50 nM), AKT constructs (125 ng for WT and M3 and 100 ng for phospho-MUT) or treated with the AKT inhibitor MK-2206 (500 nM). After 4 h from the treatments, the Incucyte Annexin V NIR Dye (Sartorius, 4768) was added to the growth media at a final concentration of 1:400 and cells maintained at 37 °C and 5% CO_2_ for up to 72 h. Phase images were acquired every 4 h (Objective: 20 X; Scan type: standard, 4 images per well; channel selection: Phase Contrast and NIR). The results were analysed using Incucyte software, where the red area (apoptotic cells)/phase area ratio was normalized to T0 for each well at each time point. Quantified time-lapse curves were generated by the software. Each experiment was performed in triplicate to ensure reproducibility.

### Quantitative real-time PCR

Total RNA was extracted from MCF7 cells buy using RNAeasy Mini Kit (Qiagen) according to the user manual. One μg of RNA was retro-transcripted by using QuantiTect® Reverse Transcription Kit (Qiagen) according to manufacturer’s instructions; cDNAs were used to perform q-PCR on Light Cycler 480 Instrument II (Roche) by using LightCycler 480® SYBR Green I Master (Roche) mix, according to manufacturer’s instructions to assess the relative abundance of AKT1 (hAKT1 fw: 5′- AGTTCTCCTACTCGGCCAG-3′; hAKT1 Rv: 5′- AATACAGATCATGGCACGAGG -3′) AKT2 (hAKT2 Fw: 5′- CCAAATTCCAGTACAGACCCAG-3′; hAKT2 Rv: TTCTAACCAAACGCTCAGGAG-3′) and AKT3 (hAKT3 Fw: CATCACCAGTCCTAGCTCTTAC-3′; hAKT3 Rv: 5′- ATGAGGGTGAAAGGTGGC-3′) transcripts. Values were normalized to the expression of reference gene GAPDH (GAPDH Fw: 5′- AGTTAAAAGCAGCCCTGGTGAC-3′; GAPDH Rv: 5′- CCACATCGCTCAGACACCAT-3′). Relative quantification analysis was conducted by using 2-ΔCt method.

### Softwares

The bioinformatic tool ADPredict, available online at www.adpredict.net, was used for the prediction of ADP-ribosylated acidic residues (aspartic and glutamic acids). Residues with the highest score were considered for subsequent mutagenesis analysis.

### Databases

The correlation between breast cancer gene expression and survival rate was analysed through the bioinformatic tool Kaplan–Meier plotter (https://kmplot.com/analysis/).

### Site-directed DNA mutagenesis and molecular cloning

Human-AKT1 E91Q, E132Q/E133/Q/E135Q, E298Q/D302N, E375Q, E397Q/D398N mutants were generated by site-directed mutagenesis reactions using the following primers:

hAKT1 E91Q Fw: 5′-CCTTCCATGTGCAGACTCCTGAGG-3′;

hAKT1 E91Q Rv: 5′- CCTCAGGAGTCTGCACATGGAAGG-3′;

hAKT1 E132Q/E133Q/E135Q Fw: 5′-GACAACTCAGGGGCTCAACAGATGCAGGTGTCCCTGGCCAAG-3′;

hAKT1 E132Q/E133Q/E135Q Rv: 5′-CTTGGCCAGGGACACCTGCATCTGTTGAGCCCCTGAGTTGTC-3’;

hAKT1 E298Q/D302N Fw: 5’-CTTCGGGCTGTGCAAGCAGGGGATCAAGAACGGTGCCACCATGAAG-3′;

hAKT1 E298Q/D302N Rv: 5′- CTTCATGGTGGCACCGTTCTTGATCCCCTGCTTGCACAGCCCAAG-3′;

hAKT1 E375Q Fw: 5′-GCACGCTTGGTCCCCAGGCCAAGTCCTTG-3′;

hAKT1 E375Q Rv: 5′-CAAGGACTTGGCCTGGGGACCAAGCGTGC-3′;

hAKT1 E397Q/D398N Fw: 5′- CTTGGCGGGGGCTCCCAGAACGCCAAGGAGATC-3′;

hAKT1 E397Q/D398N Rv: 5′-GATCTCCTTGGCGCTTCTGGGAGCCCCCGCCAAG-3′;

hAKT1 T308A Fw: 5′-GGTGCCACCATGAAGGCCTTTTGCGGCACAC-3′;

hAKT1 T308A Rv: 5′-GTGTGCCGCAAAAGGCCTTCATGGTGGCACC-3′;

hAKT1 S473A Fw: 5′-CACTTCCCCCAGTTCGCCTACTCGGCCAGCG-3′;

hAKT1 S473A Rv: 5′-CGCTGGCCGAGTAGGCGAACTGGGGGAAGTG-3′.

hAKT1 T308A Fw: 5′-GGTGCCACCATGAAGGCCTTTTGCGGCACAC-3′.

hAKT1 T308A Rv: 5′-GTGTGCCGCAAAAGGCCTTCATGGTGGCACC-3′.

hAKT1 S473A Fw: 5′-CACTTCCCCCAGTTCGCCTACTCGGCCAGCG-3′.

hAKT1 S473A Rw**:** 5′-CGCTGGCCGAGTAGGCGAACTGGGGGAAGTG-3′**.**

PCR reactions were performed using PfuTurbo Cx Hotstart DNA Polymerase (Agilent) according to the manufacturer’s instructions. The mutagenesis reaction products were cloned in pCDNA.3xHA (a gift from M. Santoro, Federico II University, Naplesi) plasmid and transformed in TOP10 chemical competent cells (Invitrogen). The constructs encoding the fusion proteins between hAKT wild-type or its mutants were cloned into the EcoRI/XhoI sites of pET28b prokaryotic expression vector (Novagen) and expressed in BL21(DE3) chemically competent cells for the expression and purification of the corresponding N-terminally His-tagged AKT proteins.

### Expression and purification of his-tagged AKT proteins from inclusion bodies

BL21(DE3) pLysS cells were transformed with plasmid encoding His-tagged AKT1(pET28dAKT1). The bacterial cultures were grown at 37 °C under continuous shaking (200 rpm), monitoring the OD600 till it reached 0.5. Bacteria were then induced with 0.3 mM IPTG at 20 °C. After ON incubation, the cultures were chilled on ice and centrifuged at 5,000 × g for 10 min at 4 °C. The pellets were subsequently resuspended in lysis buffer (10 mM Tris 7.5, 5 mM EDTA, 100 mM NaCl supplemented with protease and phosphatase inhibitors) incubated at room temperature (RT) for 20 min and then sonicated on ice in a falcon tube for 5 min (pulse: 10 s off, 30 s off, amplitude: 40%). 1% Triton X-100 was added to the homogenized lysates, and incubated on a rotor wheel for 30 min at 4 °C. Lysates were clarified by centrifugation at 10,000 rpm, 4 °C, resuspended in lysis buffer, homogenized using a small potter and furtherly centrifugated. The pellet was washed twice with wash buffer supplemented with urea (10 mM Tris 7.5, 5 mM EDTA, 5 mM DTT, 100 mM NaCl, 2% (v/v) Triton X-100, 2 mM Urea) followed by one wash in the same buffer without urea and triton. The pellet was resuspended in urea buffer (8 M Urea, 100 mM Tris 7, 5 mM EDTA, 100 mM DTT supplemented with protease inhibitor cocktail) under stirring conditions first at RT and then subsequently left overnight at 4 °C. Following day, the pellet was discarded after centrifuging for 30 min at 18,000 rpm. Concentration of the supernatant or the inclusion bodies were estimated. To refold, the inclusion bodies were diluted in dilution buffer (100 mM Tris 7.5, 5 mM EDTA, 1 mM GSH, 0.3 mM GSSG, 500 mM L-Arginine); gradually adding the buffer at a time to reach a final concentration of the protein to 200 μg/mL. The diluted inclusion bodies were left under stirring at 15 °C for ON to subsequently centrifuge at 18,000 rpm for 30 min and then the supernatant was recovered. After estimating the protein concentration, dialysis was performed against 2L dialysis buffer (100 mM Tris 7.5, 150 mM NaCl, 1 mM EDTA, 10% (v/v) Glycerol) ON and subsequently the protein was concentrated on Vivaspin 6 (with a molecular weight cut-off of 10 kDa), centrifuging for 4 hat 3,260 rpm.

### Clonogenic assays

MCF7 cells were seeded in 6-well plates (10,000 cells/ well). Following day, the cells were transiently transfected or not with PARP12 siRNAs. The cell colony growth was monitored for 10 days till the individual colonies did not merge. Subsequently, the cells were washed in PBS thrice and then fixed with 4% paraformaldehyde for 5 min. The colonies were then stained with 1% (w/v) crystal violet for 30 min. The stained colonies were by diluting them in 30% (v/v) acetic acid and collecting the cells into a 96-well plate to subsequently quantify their absorbance of crystal violet at 590 nm. Absorption measurements were normalized to the amount of crystal violet released from the control cells.

### Immunoprecipitation and immunoblotting

MCF7 cells were transiently transfected or not (Mock) with PARP12 siRNAs with Lipofectamine RNAiMAX Reagent for 48 h or cDNAs coding for HA-tagged wild-type or AKT1 point mutants’ constructs using Lipofectamine LTX and Plus reagent for 24 h.

For endogenous AKT immunoprecipitation, MCF7 cells were washed twice with ice-cold PBS 1X and lysate in CHAPS Lysis Buffer [0.3% (w/v) CHAPS, 40 mM Hepes pH 7.4, 2 mM EDTA supplemented with protease and phosphatase inhibitors]. Total lysates were passed 10 times through a 25-gauge needle with syringe, kept at 4 °C for 20 min. Lysates were clarified by centrifugation at 13,000 rpm for 10 min. Supernatants were collected and protein concentration quantified using Bradford protein assay, according to manufacturer’s instructions (Biorad). Two mg of total lysates were incubated with AKT antibody or with normal Rabbit IgG (Pre-im-IgG) on rotation for 2 h at 4 °C. Following antibody incubation, protein A Sepharose CL-4B, previously equilibrated in CHAPS Lysis Buffer, was added to the mixture and incubated on rotation for 1 h at 4 °C. The beads were washed three times with CHAPS lysis buffer supplemented with 150 mM NaCl and two times in WASH Buffer [50 mM Hepes pH 7.4, 40 Mm NaCl, 2 mM EDTA].

For endogenous p53 immunoprecipitation, MCF7 cells were washed twice with ice-cold PBS 1X and lysed in RIPA buffer [100 mM Tris–HCl pH 7.5, 1% IGEPAL (w/v), 0,5% Sodium Deoxycholate (w/v), 0,1% SDS (w/v), 150 mM NaCl supplemented with protease and phosphatase inhibitors] on rotation at 4 °C for 30 min. Lysates were clarified and quantified as described above. One or 1,5 mg of total lysates were incubated with p53 DO-1 mouse monoclonal (1 µg/mg), or with normal mouse IgG (Pre-Im-IgG) on rotation overnight at 4 °C. Following antibody incubation, protein A Sepharose CL-4B, previously equilibrated in RIPA buffer, was added to the mixture and incubated on rotation for 1 h at 4 °C. The beads were washed three times with lysis buffer and two times with tris HCl pH 7.4. The protein complexes were eluted from the beads by using SDS sample buffer. For immunoblotting, 20 μg of total cell lysate and the eluted immunoprecipitated proteins were run on SDS/PAGE and immunoblotted with primary antibodies, diluted in 5% BSA in TBS-Tween (0,1% v/v). All densitometric analysis were performed using the Image J software and normalised for total protein levels.

### Immunofluorescence and confocal microscopy

Cells were fixed with 4% paraformaldehyde for 10 min at RT, washed three times in PBS, incubated for 7 min at RT in 0,1% Triton in PBS and then incubated for 30 min at RT in blocking solution (0.5% bovine serum albumin, 50 mM NH_4_Cl in PBS, pH 7.4, 0.1% saponin and 0.02% sodium azide). Cells were subsequently incubated with the indicated antibodies diluted in blocking solution for ON at 4 °C. After incubation with the primary antibody, cells were washed three times in PBS and incubated with a fluorescent-probe-conjugated secondary antibody, for 30 min at RT. Alexa Fluor 488-, 568- or 647 conjugated anti-rabbit, anti-mouse or anti-goat donkey antibodies were used at a dilution of 1:400 in blocking solution. After immunostaining, cells were washed three times in PBS and twice in sterile water. The coverslips were then mounted on glass-microscope slides with Mowiol. Images were taken using a Zeiss-LSM 980 confocal microscope. Optical confocal sections were taken at 1 Air Unit. Quantitative analysis was performed using the Image J software. In brief, for γH2AX, it was measured the percentage of cells showing nuclear staining. For p53 nuclear staining, the Nuclear/Cytoplasmic ratio was calculated by measuring the relative fluorescence integrated intensities in the nuclear and cytoplasmic areas. The cytoplasmic area was defined using AKT staining, which served as a marker for the total cell area.

### Proximity ligation assay

Proximity ligation assays were performed using the Duolink anti-goat MINUS (DUO92006) and anti-Rabbit PLUS (DUO92002) in situ PLA probes and the Duolink in situ Detection Reagents Red (Sigma-Aldrich), following the manufacturer’s instructions. The amplified signals were acquired using laser scanning confocal microscopy and analysed with the Image J software. In brief, the PLA puncta were identified as objects with a signal above a minimum threshold and quantified as number of PLA puncta/cell.

### Af1521 *macro* domain-based pull-down assay

MCF7 were transiently transfected or not to follow the ADP-ribosylation of overexpressed HA-tagged or endogenous AKT. Post transfection, cells were lysed or solubilized following the same protocol described above. The supernatants were recovered, and the protein concentration was evaluated using the BCA Protein Assay Kit (Thermo Fisher Scientific). The total cell lysates (1–1.5 mg) were further incubated with 46 μg GST-tagged- macro domain, as previously described [5, 20].

### In vitro ADP-ribosylation assay

One μg of purified His-tagged, full-length AKT1 (wild-type or relative point mutants) was incubated with 250 ng GST-tagged catalytic fragment of GST-tagged PARP12 in ADP-ribosylation buffer (50 mM Tris–HCl pH 7.4; 4 mM DTT; 500 μM MgCl_2_; 100 μM NAD^+^), at 37 °C for 60 min. At the end of the incubation, the reaction was stopped by adding SDS sample buffer.

### GST-pull down assay

GST pull-down assays were carried out as previously described [21], with some modifications. Briefly, 3 μg of purified His-tagged AKT were incubated with 300 ng GST-tagged PARP12 catalytic fragment in ADP-ribosylation buffer at 37 °C for 60 min. Then, 30 μl glutathione sepharose beads were added in GST incubation buffer (50 mM Tris, pH 8.0, 100 mM NaCl, 0.2% Triton X-100, protease inhibitors) for a further incubation on rotation for 1 h at 4 °C. The beads were then washed three times with GST incubation buffer, recovered by centrifugation (750* g*, 5 min at 4 °C) and further washed two times with the same buffer without Triton X-100. The bound protein was eluted from the glutathione sepharose beads with GST elution buffer (100 mM Tris, pH 8.0, 20 mM glutathione, 5 mM dithiothreitol). The 10% and 80% of the input and eluted proteins, respectively, were processed for 8% SDS/PAGE and western blotting analysis.

### Statistics and reproducibility

All of the quantified western blots are the mean ± SEM or mean ± SD, as indicated in figure legends, of at least three independent experiments. Quantitative analysis was performed using the Image J software. For statistical analysis, p-values were calculated from at least three experiments, comparing control and each treated group individually using Student’s t-test. All statistical parameters are listed in the corresponding figure legends. For all statistical tests, P < 0.05 was considered significant and is indicated by asterisks. For vitality assays, One-way ANOVA multiple comparisons test and Student’s t-test were applied using the GraphPad Prism 7 software. P-values < 0.05 were considered significant.

## Results

### PARP12 depletion triggers apoptosis in a subset of breast cancer cell lines

**Fig. 1:**
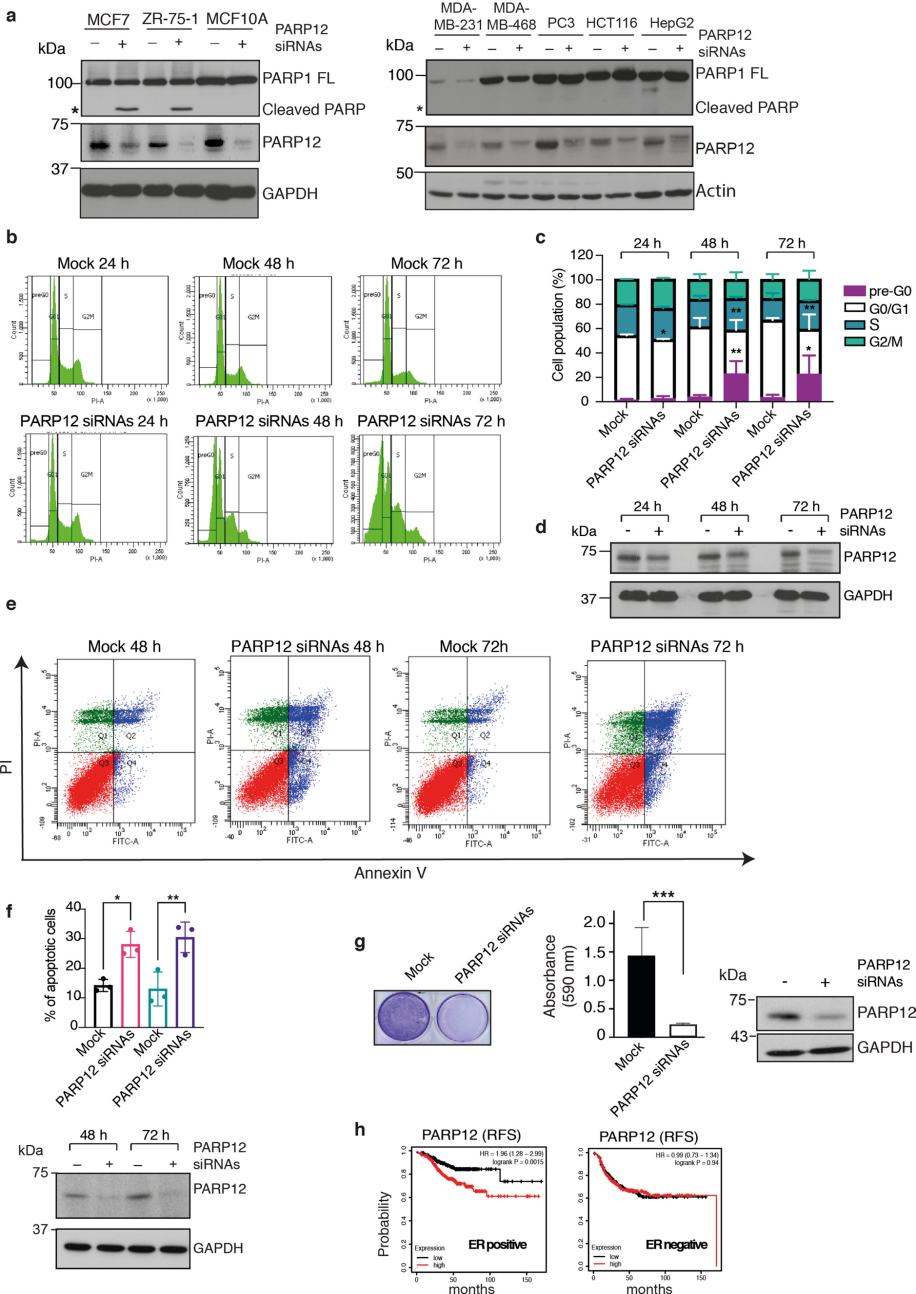
PARP12 depletion induces apoptosis in ER + -breast cancer cell lines. **a** Representative western blotting analysis of lysates from different cell lines (as indicated) transfected or not with PARP12 siRNAs for 72 h. PARP1, PARP12, GAPDH and actin signals were detected (as indicated). **b** MCF7 cells transfected or not (Mock) with PARP12 siRNAs for 24 h, 48 h and 72 h (as indicated) were subjected to cell-cycle analysis by flow cytometry using propidium iodide (PI) staining. **c** Quantification of the FACS analysis as reported in (**b**). Data are the mean of three independent experiments ± SD. *P < 0.05, **P < 0.01 *versus* mock (one-way ANOVA with Sidak's multiple comparisons test). **d** Representative western blotting analysis of PARP12 depletion levels of MCF7 cells subjected to cell-cycle analysis (as reported in b.). GAPDH used as loading control. **e** MCF7 cells were stained with Annexin V/PI and analysed by FACS after 24 h, 48 h and 72 h of transfection with PARP12 siRNAs (as indicated). **f** Top: Quantification of Annexin V-positive apoptotic cells reported in (**e**) (see “Methods”). Graphs refer to cell populations in the Q2 (upper-right quadrant) and Q4 (lower-right quadrant) regions of the flow cytometry scatter plot. Data are the mean of three independent experiments ± SD. *P < 0.05, **P < 0.01 *versus* mock (one-way ANOVA with Sidak’s multiple comparisons test). Bottom: Analysis of PARP12 depletion levels in MCF7 cells subjected to apoptosis analysis (as reported in **e**). **g** Left panel: Representative images of crystal violet staining of MCF7 cells in the colony formation assays after 10 days transfection with PARP12 siRNAs (see Methods). Middle panel: Quantification of the Stained colonies dissolved in 33% acetic acid by measuring the absorbance at 590 nm. Data are mean ± SEM of three independent experiments performed in triplicate ***P ≤ 0.001 *versus* mock (Student’s t-tests). Right panel: Analysis of PARP12 depletion levels in MCF7 cells subjected to clonogenic assay. GAPDH used as loading control. Molecular weight standards (kDa) are indicated on the left of each panel. **h** Kaplan–Meier plot showing inverse correlation between PARP12 levels and relapse-free survival (RFS) in ER + breast cancers, over a period of 150 months. No effect observed in ER negative breast cancer patients

As mentioned before, PARP12 has been identified as a key factor in causing breast cancer resistance to genotoxic stress [18]. Based on this evidence, to understand the molecular mechanism underlying this phenotype, we investigated the impact of PARP12 depletion on apoptosis induction on a broad set of cancer cell lines of varied tumour origin. In brief, a pool of PARP12 siRNAs was transfected in the different cell lines (as indicated in Fig. 1a) for 72 h and apoptosis induction followed by detection of PARP1 cleavage, a recognised marker of caspase-dependent apoptosis [22]. As shown in Fig. 1a, absence of PARP12 specifically induced apoptosis in a subset of breast cancer cells, harbouring the ER expression (*i.e.* MCF7 and ZR-75–1) whilst not affecting the non-tumorigenic epithelial cell line MCF10A; in addition, no effect was observed in the other cell lines tested (triple negative breast cancer MDA-MB-231 and MDA-MB-468 cells, prostate cancer PC3 cells, colon cancer HCT116 cells and hepatocarcinoma HepG2 cells), suggesting a specificity of PARP12 in cell survival of ER + -breast tumour models. MCF7 cells have been extensively used in breast cancer literature as a model system therefore this cell line was selected for all subsequent analyses.

MCF7 cells were analysed for cell-cycle progression under PARP12 depletion conditions. Cells were stained with propidium iodite to follow the cell-cycle distribution by flow cytometry at 24 h, 48 h and 72 h post PARP12 siRNA transfection. As shown in Fig. 1b, control cells (Mock) showed a typical DNA pattern distribution in G0/G1, S and G2/M phases of the cell cycle, whilst MCF7 cells showed the appearance of the sub-G0 phase upon knock-down of PARP12 at 48 h and 72 h that is typically present in cells undergoing apoptosis (Fig. 1b–d).

Apoptosis induction was evaluated by Annexin-V staining using FACS analysis. Plots and relative quantifications reported in Fig. 1e, f showed an increase of about 20% of apoptotic cells upon PARP12 depletion, in line with the data reported above. As further confirmation, colony assays (see methods) performed on MCF7 cells demonstrated that PARP12 silencing strongly interfered with survival and growth of MCF7 cells (Fig. 1g).

Importantly, we also performed a meta-analysis, exploiting the Kaplan–Meier Plotter (https://kmplot.com/analysis/); this is a large public database containing gene expression datasets from cancer populations with associated clinical data [23], that was used to investigate how PARP12 gene expression affected the survival of breast cancer patients. The analysis was peformed on a cohort of patients following systemic treatment (chemotherapy and endocrine therapy) that show both positive and negative expression of the oestrogen receptor. As the Fig. 1h left panel shows, higher PARP12 expression (in red) correlated to decreased relapse-free survival (reported on the Y-axis as probability) specifically in ER + breast cancer patients. Instead, in ER-negative breast cancer patients, no differences were observed (Fig. 1h, right panel).

Overall, these data demonstrate a role for PARP12 in cell survival of a subset of ER + breast cancer cells, supporting an involvement of PARP12 in drug-resistance acquisition.

### AKT is MARylated by PARP12

Based on the above data, we reasoned that the effect on apoptosis induction observed in breast cancer cell lines could be associated with the ability of PARP12 to ADP-ribosylate target proteins required for cell survival. Interestingly, according to Gaston and colleagues and in line with Kaplan-Maier analysis (Fig. 1h), PARP12 has the potential to act as a key factor in the acquisition of drug-resistance mechanisms [18]. Alteration in the AKT pathway has often been associated with the development of resistance processes [1]; therefore, we focused on a possible inter-connection between PARP12-mediated MARylation and the AKT pathway.

**Fig. 2:**
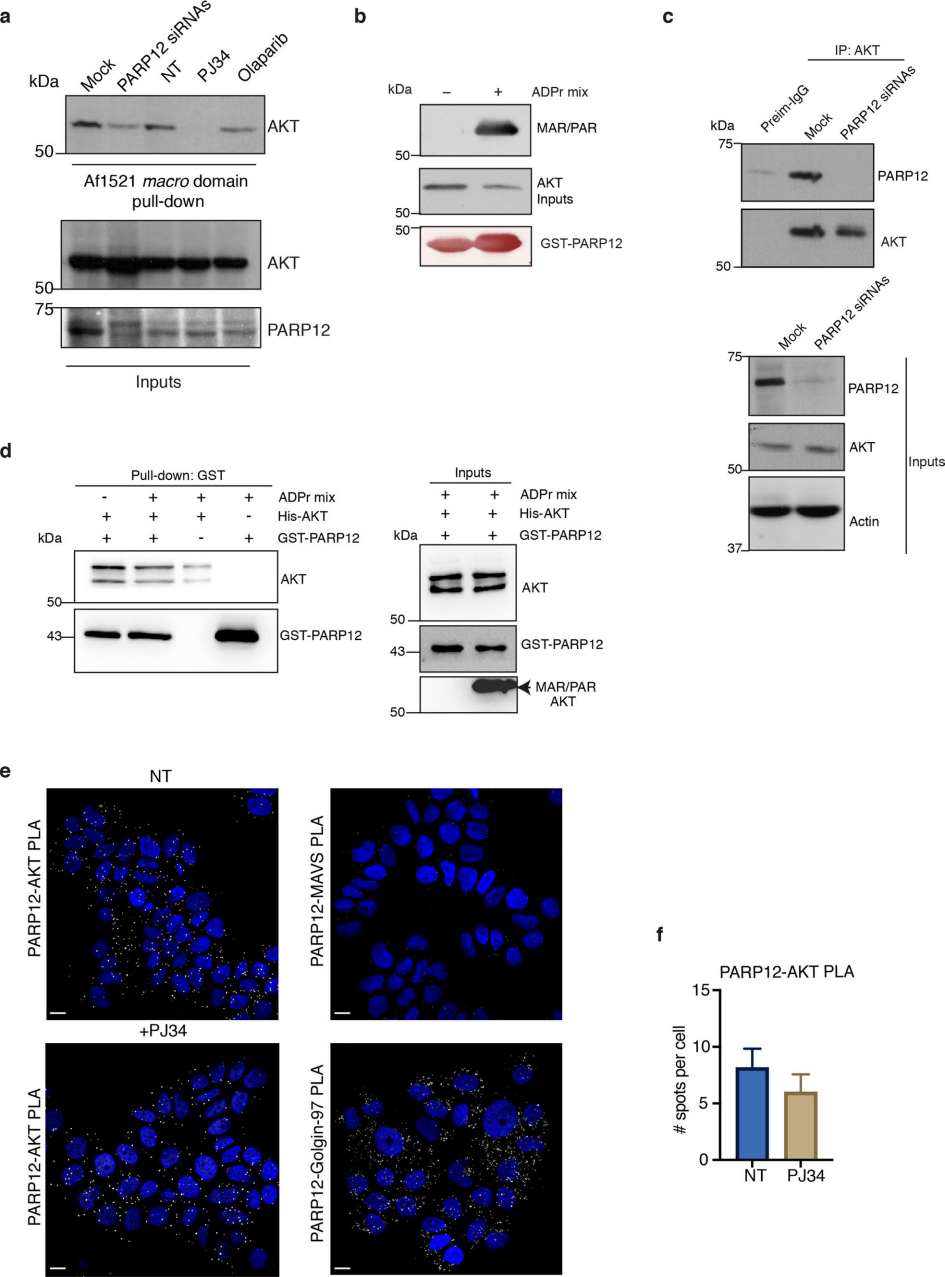
AKT is a substrate of PARP12-catalysed MARylation. **a** Af1521 *macro* domain–based pull-down assay of total cell lysates from MCF7 cells transfected or not (Mock) with PARP12 siRNAs for 72 h or treated with the PARP inhibitor PJ34 (50 µM, 2 h), or with PARP1/2 inhibitor Olaparib (30 μM, 2 h), showing the PARP12-dependent MARylation of AKT. **b** In vitro ADP-ribosylation assay using GST-tagged purified PARP12 catalytic fragment (250 ng) and His-tagged purified AKT1 (1 μg), in the presence of 100 μM NAD^+^, detected by using an antibody for MARylated or PARylated proteins (MAR/PAR). PARP12 total levels detected by Ponceau S staining. **c** Endogenous AKT immunoprecipitation (IP: AKT) from lysates of MCF7 cells transfected or not with PARP12 siRNAs for 48 h. Representative western blotting (antibodies as indicated) of total lysates (inputs) and immunoprecipitated proteins with pre-immune-IgG (Preim-IgG) or anti-AKT-IgG. **d **In vitro GST pull-down assay using His-tagged purified AKT1 (3 μg) and GST-tagged purified PARP12 catalytic fragment (300 ng), previously subjected to an in vitro MARylation assay, as indicated above. Bound proteins were eluted and detected by Western blotting with an anti–AKT and an anti-GST antibodies. ADP-ribosylation was detected by using an antibody for MARylated or PARylated proteins (MAR/PAR). Molecular weight standards (kDa) are indicated on the left of each panel. **e** Representative confocal images of in situ proximity ligation assay (PLA) between PARP12 and AKT in MCF7 cells were treated or not with PARP inhibitor PJ34 (50 µM, 2 h). White dots indicate the proximity of PARP12 and AKT. PLA negative control with MAVS-PARP12 antibodies and PLA positive control with Golgin-97-PARP12 are also shown. Scale bar 10 µm. Quantification is reported in (**f**). n = 100 cells from three different experiments, ± SD; ***P < 0.001, calculated by Student’s t test (not significant is not reported)

Consequently, we investigated the possibility of AKT to be the target of ADP-ribosylation in MCF7 cells and evaluated its ADP-ribosylation under different experimental conditions. MCF7 cells were transiently transfected with a PARP12 siRNA pool or treated with the general PARP inhibitor PJ34 and ADP-ribosylated proteins were recovered from total cell lysates by performing the Af1521 *macro* domain based pull-down assay, a well-recognized method to detect ADP-ribosylated proteins [20, 24]. Cells treated with the PARP1/2 inhibitor Olaparib were used as control. The ADP-ribosylated pool of AKT (*i.e.* the pool of AKT bound to the Af1521 *macro* domain) was detected using an AKT specific antibody. Figure 2a shows that AKT was ADP-ribosylated in mock or untreated (NT) cells; importantly, AKT binding to the Af1521 *macro* domain was reduced (up to 80%) upon PARP12 depletion, accounting for the specificity of PARP12 in catalysing this reaction. The same effect was observed upon treatment with the general PARP inhibitor PJ34, but not when the PARP1/2 selective inhibitor Olaparib was used, further supporting the specificity of PARP12 in catalysing AKT MARylation.

AKT MARylation was then analysed in in vitro assays, using purified full-length His-tagged AKT1 and GST-tagged PARP12-catalytic domain, in the presence of NAD^+^; ADP-ribosylated AKT was detected by using a specific antibody that recognizes ADP-ribosylated proteins (MAR/PAR). As reported in Fig. 2b, AKT was modified under these conditions, indicating that it is a specific, direct substrate of PARP12-dependent ADP-ribosylation. In line with this data, co-immunoprecipitation experiments were able to detect an interaction between PARP12 and AKT at the endogenous level (Fig. 2c), further supporting the role of PARP12 in the regulation of AKT functions. To assess whether this interaction is direct and dependent on MARylation, we performed in vitro assays with purified recombinant proteins, in the presence or absence of the ADP-ribosylation mix. Specifically, GST-tagged PARP12 and His-tagged AKT1 were incubated with or without the ADP-ribosylation mix, and then GST-PARP12 was recovered and eluted using reduced glutathione. The efficiency of ADP-ribosylation reaction was checked using a MAR/PAR antibody on the input fraction. As shown in Fig. 2d, the two proteins interact directly, regardless of ADP-ribosylation. Consistent results were observed in MCF7 cells using proximity ligation assay (PLA), which detected the PARP12-AKT interaction under native conditions (Fig. 2e, f). Treatment with the PARP inhibitor PJ34 did not alter PLA signals, validating the in vitro data and confirming that the interaction between PARP12 and AKT is independent of MARylation.

### Identification and validation of MARylated residues in AKT

AKT exists as three different isoforms, namely AKT1, AKT2 and AKT3; despite their high similarity (80%), the distinct AKT isoforms exert non-redundant functions, partially even opposing effects under physiological and pathological conditions [25].

Based on this knowledge, we analysed AKT isoforms in our cellular model by qRT-PCR; as shown in Fig. S1a, AKT1 was the main isoform to be expressed in MCF7 cells, therefore we focused on AKT1 for bioinformatic and mutagenesis experiments.

**Fig. 3:**
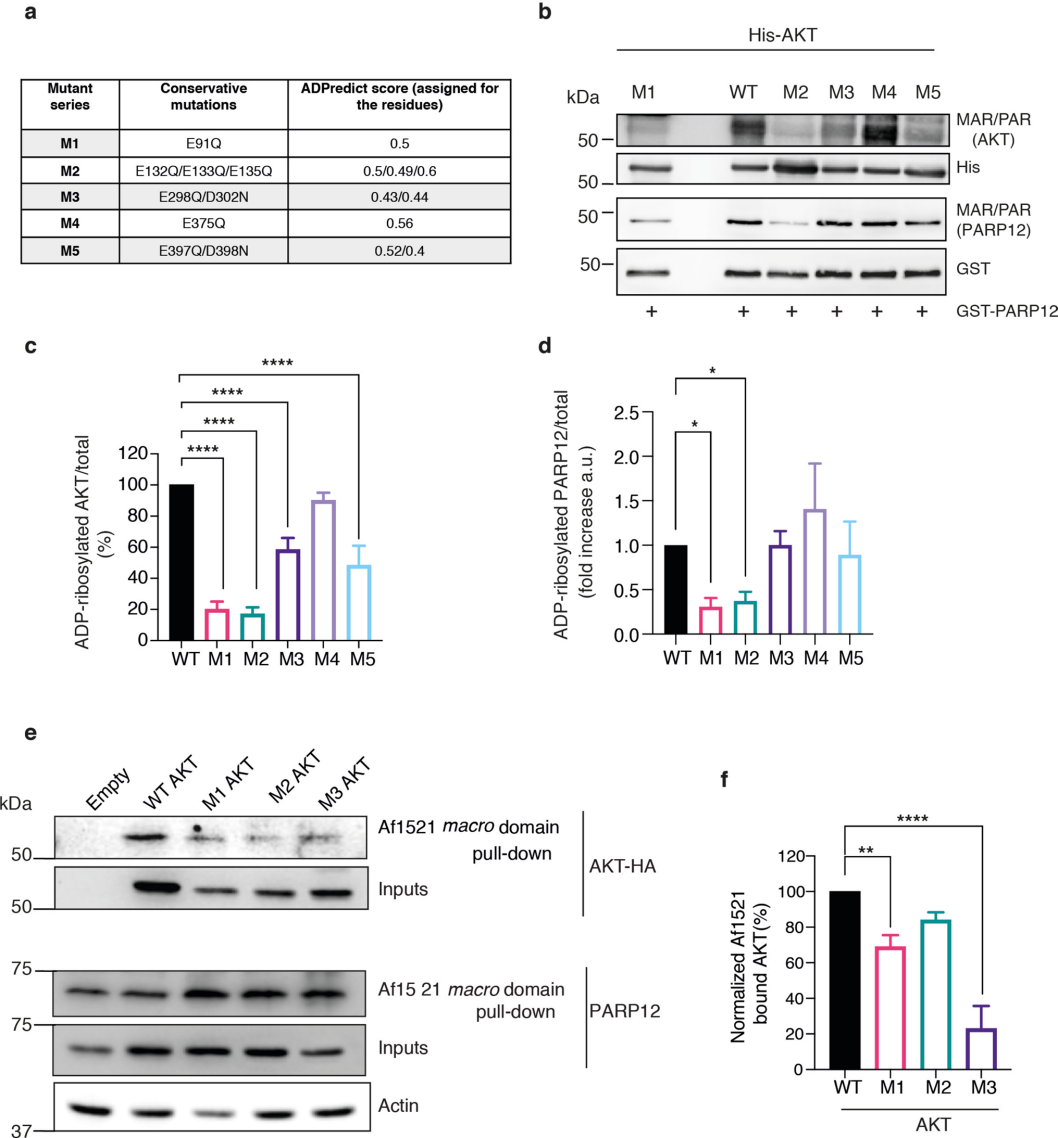
Identification and validation of AKT1 residues modified by PARP12. **a** Table of AKT1 point mutants generated based on the ADP-ribosylated residues identified by the ADPredict tool. Prediction scores assigned to the individual AKT residues are also reported. **b** Representative western blotting analysis of in vitro ADP-ribosylation assays performed using GST-tagged purified PARP12 catalytic fragment and His-tagged purified AKT1 WT or relative point mutants (M1-M5), in the presence of 100 μM NAD^+^. ADP-ribosylated proteins were detected by using an antibody against ADP-ribose (MAR/PAR); total AKT and PARP12 levels were monitored with anti-His antibody (His-AKT) and GST-antibody, respectively. Quantifications ± SD of MARylated AKT (**c**) and PARP12 (**d**) normalized on total protein levels are reported. *P < 0.05 and ****P < 0.0001 *versus* WT AKT (one-way ANOVA with Dunnett’s multiple comparisons test). **e** Af1521 *macro* domain–based pull-down assay of total lysates of MCF7 cells transfected with HA-tagged AKT1 WT or its point mutants (M1-M3, as indicated). The bound proteins were eluted and detected by western blotting with an anti–HA antibody. **f** Quantifications ± SD of Af1521 bound-AKT normalized on total AKT levels. **P < 0.01, ****P < 0.0001 *versus* WT AKT (one-way ANOVA with Dunnett’s multiple comparisons test)

Built on the evidence that PARP12 modifies acidic amino acid residues [5, 17, 26], we applied the bioinformatic tool ‘ADPredict’, designed to identify the putative acidic amino-acid residues of target proteins most prone to MARylation [27]. According to prediction score, 5 regions were selected as major targets of MARylation and conservative point mutations were introduced to generate full-length AKT1 variants with the following point mutations: M1 (E91Q), M2 (E132Q/E133Q/E135Q), M3 (E298Q/D302N), M4 (E375Q), and M5 (E397Q/D398N), as detailed in the table in Fig. 3a. As a first approach of validation, AKT1 mutants were tested for ADP-ribosylation levels in in vitro assays. In vitro ADP-ribosylation reactions were performed using the His-tagged recombinant AKT1 proteins (wild-type and AKT1 point mutants M1-M5, as indicated) and the GST-tagged catalytic fragment of PARP12, in the presence of NAD^+^. Following the reactions, proteins were processed for western blotting analysis, using the antibody specific for ADP-ribosylated proteins (MAR/PAR, Fig. 3b). Here, the AKT MARylation levels were normalised with total AKT protein probed with histidine antibody. MARylation of PARP12 was detected as internal positive control of the reaction. As shown in the Fig. 3 b and 3c, the data revealed all the analysed mutants to show defects in MARylation levels relative to the wild-type (WT) except for M4. Mutants M1 and M2 showed the least binding to the Af1521 *macro* domain, with only 13.6% and 15.6% MARylated fractions relative to the WT. M5 and M3 followed, with Af1521 binding fractions of 51.6% and 62.3%, respectively. All values were quantified as the MARylated fraction relative to the WT, which was set at 100%. On the other hand, M4 failed to display a defect in the modification with an of average of 94.3% modified proteins. Unexpectedly, PARP12 MARylation levels were significantly decreased in the presence of M1 and M2 (Fig. 3b, d). This indicates that M1 and M2 inhibit PARP12 catalytic activity, and the observed reduction in AKT MARylation is likely a consequence of this inhibition rather than being solely attributed to mutations in AKT itself. Based on these findings and to further investigate the role of the identified amino acid residues, we excluded M4 from subsequent validation analyses in cells, while retaining the other mutants (M1, M2, M3, and M5) for further study.

The selected mutants were thus analysed for their ADP-ribosylation levels in cells, upon over-expression of HA-tagged constructs in MCF7 cells. All mutants could be over-expressed in MCF7 cells except mutant M5, that therefore was not included for subsequent analyses. Af1521 *macro* domain-based pull-down assays were performed on total MCF7 lysates obtained from cells transiently transfected with the plasmids coding for HA-tagged wild-type AKT1 or relative mutants (*i.e.* M1, M2, M3). Cells transfected with the empty vector were used as control. The ADP-ribosylated pool of AKT was detected using HA-antibody that specifically recognises the over-expressed proteins. The data shown in Fig. 3e, f indicate that relative to WT, M3 exhibited the least binding to *macro* domain (an average of 25% bound to *macro* domain), followed by M1 (69%), whilst no significant reduction was observed for mutant M2; under these conditions, PARP12 MARylation remained unchanged, confirming that the reduced MARylation observed for AKT M3 is specific.

**Fig. 4:**
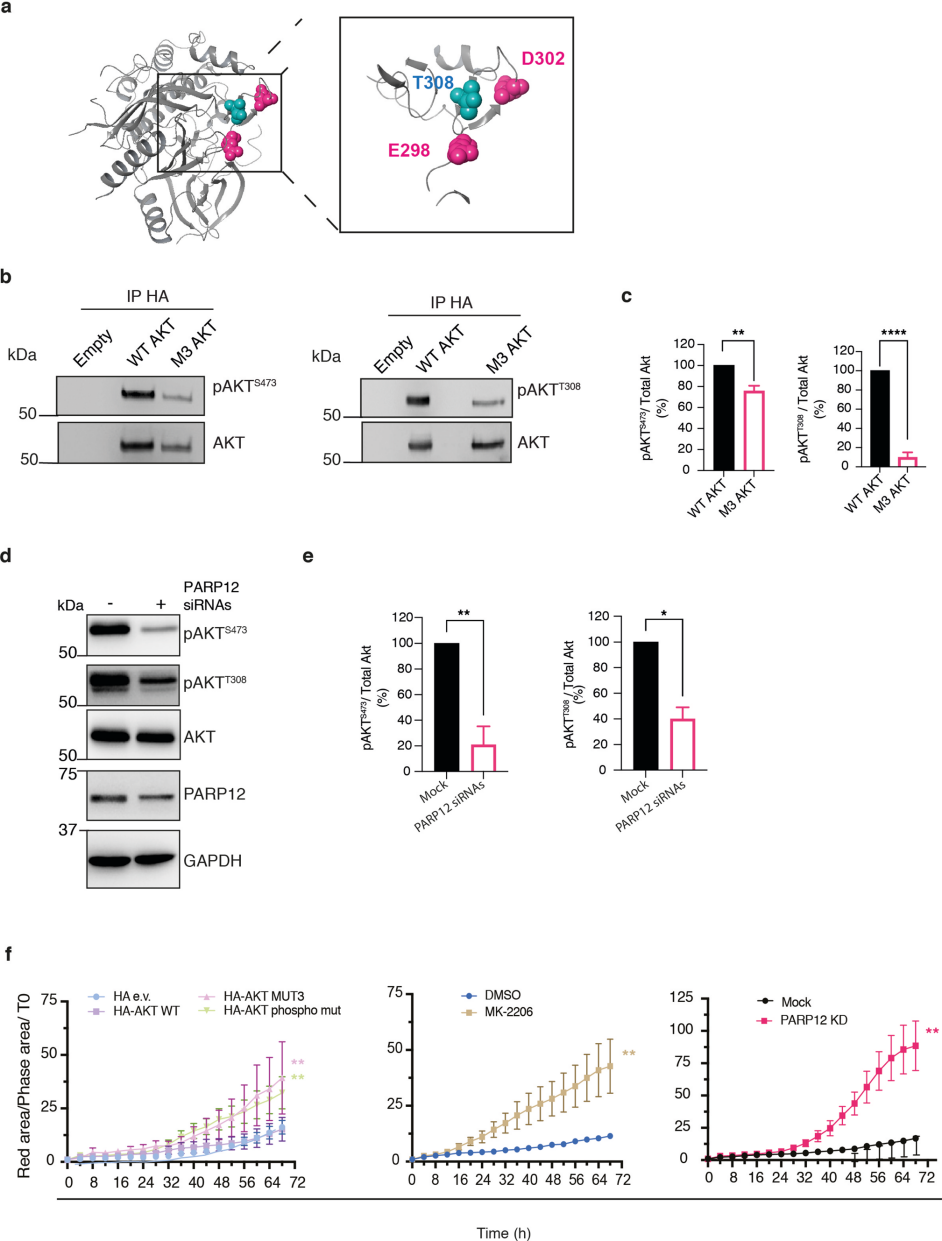
MARylation of AKT is required for its activation by upstream kinases. **a** Crystal structure of human AKT1 (PDB ID 6HHJ). Validated ADP-ribosylated residues E298 and D302 are reported in magenta; residue T308 is in cyan and has been reported as main AKT phosphorylation site. **b** Immunoprecipitation of HA-tagged AKT (IP: HA) from MCF7 cells transfected with WT HA-tagged AKT (WT AKT) or its ADP-ribosylation defective mutant M3 (M3 AKT); cells transfected with the empty vector were used as control. Representative western blotting of phosphorylated AKT fractions and total AKT are shown (pAKT^S473^, pAKT^T308^, AKT) (**c**). Quantification of AKT phosphorylation levels. Data are the mean of three independent experiments ± SD. **P < 0.01, ***P < 0.001, calculated by Student’s t test. **d** Representative western blotting analysis of endogenous AKT phosphorylation levels (pAKT^T308^, pAKT^S473^) of MCF7 cells transfected or not with PARP12 siRNAs for 72 h. Total AKT, PARP12 and GAPDH protein levels are also shown. Molecular weight standards (kDa) are indicated on the left of each panel. Data are representative of three independent experiments. **e** Quantifications ± SD are reported in the graphs. *P < 0.05 and **P < 0.01 *versus* mock (one-way ANOVA with Sidak's multiple comparisons test). **f** Time-course analysis for the effects of PARP12 depletion, AKT inhibition or expression of AKT ADP-ribosylation defective mutants on cell death. MCF7 cells were **a** transiently transfected with HA-tagged AKT WT, ADP-ribosylation defective mutant (MUT3) or with a phosphorylation mutant (T308A/S473A, phospho mut), **b** treated with AKT inhibitor MK-2206 (500 nM) or **c** transiently transfected with PARP12 siRNAs. After 4 h, Annexin V NIR dye was added to the culture media (1 μg/ml), and cells were scanned every 4 h (4 frames per well) for 72 h at the incucyte system, to follow apoptosis induction. Graphs show annexin V fluorescence as mean ± SEM, from three different experiment (ratio of red area/phase area normalised to T0). **P < 0.01 *versus* AKT WT or mock (one-way ANOVA with Sidak’s multiple comparisons test)

Collectively, this set of data indicate that residues 298 and 302 (mutated in M3) are the main targets for MARylation in cells. From a structural point of view (Fig. 4a), these two residues result as being in close proximity with threonine 308, suggesting a potential involvement in the regulation of AKT phosphorylation and activation. We therefore concentrated on mutant M3 (E298Q/D302N) for further analyses.

### MARylation of AKT is required for its activation by upstream kinases to preserve cell survival

Having defined the modification sites of AKT, we tested if MARylation could perturb AKT phosphorylation, hence activation and its functions. AKT is a serine/threonine kinase, whose activation mainly relies on two phosphorylation residues, the threonine 308 (T308) and the serine 473 (S473). Once activated, AKT exerts antiapoptotic effects through phosphorylation of substrates that directly regulate the apoptotic machinery [28].

To examine the role of MARylation in the regulation of AKT phosphorylation, we initially tested the phosphorylation status of AKT1 on both T308 and S473 upon over-expression of the AKT ADP-ribosylation defective mutant M3 (Fig. 4). Thus MCF7 cells were transfected with constructs coding for the HA-tagged wild-type and mutant M3 AKT. After 24 h, the over-expressed proteins were immunoprecipitated with an HA antibody and the samples analysed using phospho-AKT antibodies (pAKT^T308, S473^) or HA antibody, to detect phosphorylated and total levels of immunoprecipitated AKT, respectively. As represented in Fig. 4b, c, the results demonstrate a significant decrease in the phosphorylation levels of both residues. Whilst the fraction of phosphorylated mutant protein on the S473 residue was reduced to 70%, the phosphorylation level on T308 residue was almost abolished with just 10% relative to the wild-type counterpart (Fig. 4c). Differently, M1 and M2 AKT mutants did not show reduction in phosphorylation levels (Fig. S1b), corroborating the importance of MARylation in the regulation of AKT activation.

Similar results were observed for endogenous AKT upon PARP12 depletion (Fig. 4d, e). AKT phosphorylation levels were also analysed in other breast cancer cell lines, both ER + (ZR-75–1) and ER- (MDA-MB-231). A consistent reduction in pAKT levels was observed in the ER + ZR-75–1 cell line, while ER- MDA-MB-231 cells did not exhibit a similar decrease in phosphorylation upon PARP12 depletion (Fig. S2). These findings further support the involvement of AKT down-regulation in the PARP12-dependent apoptotic pathway, particularly in PARP12-sensitive cell models.

To specifically investigate the role of MARylated AKT in the observed apoptotic phenotype, apoptosis induction was monitored over 72 h via real-time Annexin V staining (see “Methods”) under the following conditions:

(i) MCF7 cells over-expressing wild-type AKT or its mutants (M3 and the phosphorylation defective-mutant T308A/S473A);

(ii) MCF7 cells treated with the AKT inhibitor MK-2206 (500 nM);

(iii) MCF7 cells depleted of PARP12 expression, used as a positive control.

As shown in the graph in Fig. 4f, over-expression of the ADP-ribosylation-defective mutant M3 induced apoptosis, highlighting the role of MARylation in the observed phenotype. Similar results were obtained with AKT inhibition and over-expression of its phosphorylation-defective mutant. Taken together, our results indicate the direct involvement of AKT in apoptosis and underscore the critical role of active, MARylated AKT in this pathway.

### PARP12 depletion correlates with increased DNA-damage, p53 nuclear translocation and FOXO1 pro-apoptotic activity

Data herein reported show that PARP12-mediated MARylation is required for AKT phosphorylation by upstream kinases, hence for its activation; consequently, absence of PARP12 may impact on AKT-downstream signalling, causing apoptosis.

**Fig. 5:**
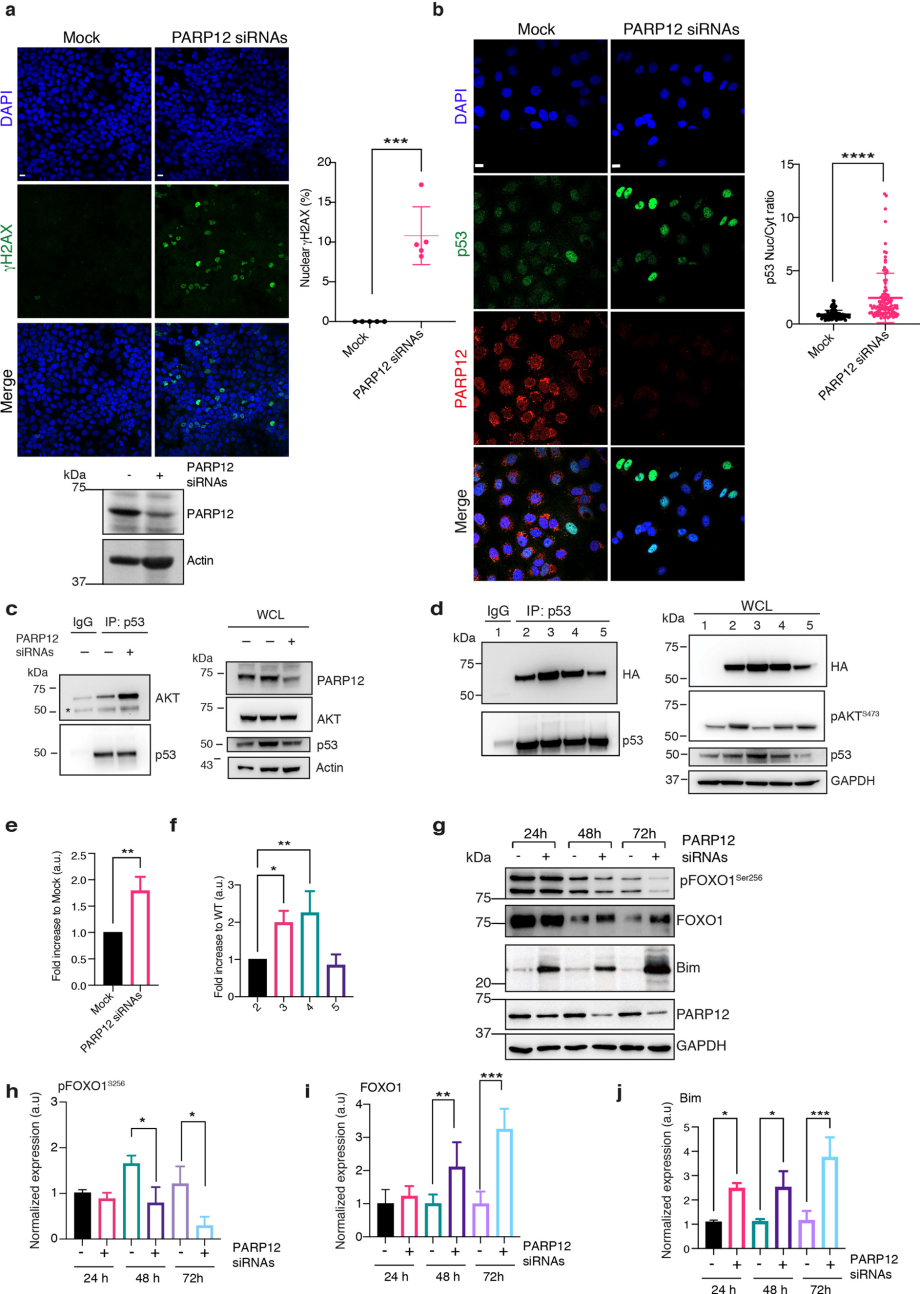
PARP12 depletion correlates with an increased DNA-damage, p53 nuclear translocation and upregulation of pro-apoptotic proteins. **a** Representative confocal microscopy images of MCF7 cells depleted or not of PARP12 expression for 72 h and stained with the DNA damage marker γ-H2AX (green). Graph on the right shows the quantification of cells with γH2AX nuclear localization. Bottom: analysis of PARP12 depletion level. Actin is used as loading control. Molecular weight standards (kDa) are indicated on the left of each panel **b** Representative confocal images of MCF7 cells transfected or not with PARP12 siRNAs for 72 h and stained for endogenous p53 (green) and PARP12 (red). Quantification of the p53 nuclear/cytoplasmic ratio (Nuc/Cyt Ratio) is reported. Nuc/Cyt Ratio was calculated based on fluorescence intensities of the signal in each compartment, see Methods. N = 100 cells from three different experiments, ± SD; ***P < 0.001, calculated by Student’s t test (scale bars, 10 µm). **c** Co-immunoprecipitation of endogenous p53 and AKT from MCF7 cells transfected or not with PARP12 siRNAs for 48 h. Inputs were also monitored as indicated. **d** Co-immunoprecipitation of endogenous p53 and HA-tagged AKT from MCF7 cells transfected with HA-empty vector (# 1) or AKT WT (# 2) or AKT phospho-MUT (# 3) or AKT M3 (#4) or AKT M1 (# 5). Inputs were also monitored as indicated. **e**, **f** Quantifications relative to c. and d., respectively. ± SD are reported in the graphs. *P < 0.05 and **P < 0.01, *versus* mock or AKT WT (one-way ANOVA with Sidak's multiple comparisons test). **g** Representative western blotting of the indicated proteins in MCF7 cells transfected or not with PARP12 siRNAs for 24 h, 48 h and 72 h. GAPDH is shown as loading control; molecular weight standards (kDa) are indicated on the left of each panel. Data are representative of three independent experiments. **h**–**j** Quantifications relative to (**d**). ± SD are reported in the graphs. *P < 0.05, **P < 0.01 and ***P < 0.001 *versus* mock (one-way ANOVA with Sidak’s multiple comparisons test)

Based on the apoptosis induction occurring upon PARP12 knock-down (Fig. 1), we evaluated DNA-damage under the same experimental conditions, by immunofluorescence analysis of cells stained with an antibody specific for the phosphorylated pool of histone H2AX on Ser 139 (γH2AX), a recognised marker of DNA damage [29, 30]. Figure 5a shows that absence of PARP12 selectively correlated with formation of γH2A.X foci, suggesting an interconnection with DNA-damage repair mechanisms. Concerning this result, it is worth mentioning the existence of a nuclear PI3K–AKT complex involved in attenuating drug-induced DNA-damage and FOXO-regulated cell death, in a way that depends on p53 for the formation of the complex. Specifically, p53 controls nuclear AKT activation, by fostering the assembly of an active AKT/PDK1/mTORC2 complex [31].

Our data show that lack of PARP12 impairs AKT activation and induces a DNA-damage dependent apoptosis. Therefore, we sought to evaluate p53 subcellular localisation, as readout of p53 activation, and AKT-p53 interaction, upon PARP12 depletion, to clarify a possible interdependence between PARP12-catalysed MARylation and AKT-p53 pathways. MCF7 cells were transiently transfected with a PARP12 siRNA pool for 72 h and endogenous p53 subcellular localisation followed by confocal microscopy analysis. Figure 5b shows an increase in p53 nuclear staining upon PARP12 depletion (measured as nuclear/cytoplasmic ratio, see methods), in line with p53 involvement in DNA-damage response.

As previously mentioned, AKT and p53 can directly interact and their binding linearly increases as a function of drug-induced DNA-damage to favour activation of DNA repair mechanisms [31]. Based on this evidence and on our data, we evaluated the p53-AKT complex in the absence of PARP12, a condition that leads to DNA-damage (see Fig. 5a). Figure 5c shows that the p53-AKT interaction was enhanced in the absence of PARP12, as detected by co-immunoprecipitation experiments with endogenous proteins (Fig. 5c, e). To specifically evaluate the role of AKT MARylation in this interaction, we performed co-immunoprecipitation experiments to assess the interaction between endogenous p53 and over-expressed AKT wild-type or ADP-ribosylation mutants M3 and M1. As an additional control, we included the phosphorylation-defective AKT mutant (T308A/S473A). As shown in Fig. 5d, both M3 (line 4) and the phosphorylation-defective mutant of AKT (line 3) exhibited stronger interactions with p53 (Fig. 5f), similar to the results observed under PARP12-depletion conditions (Fig. 5e). No effect was observed with AKT M1 (line 5), as expected, since it is not defective in ADP-ribosylation and phosphorylation (see Fig. 3 and Fig. S1b).

When analysed in light of the apoptotic effect observed under impaired MARylation conditions (*i.e.*, PARP12 knock-down and over-expression of the ADP-ribosylation-defective mutant M3, Fig. 4f), the interaction data suggest that although AKT is present in a more stable complex, its inactive form is unable to mitigate the basaline DNA damage, thereby promoting prolonged DNA damage and triggering the apoptotic cascade.

To clarify p53's role in the PARP12-dependent phenotype, we evaluated apoptosis by monitoring PARP1 cleavage and DNA damage using the γH2AX marker, in MCF7 cells depleted of both PARP12 and p53 (Fig. S3). No difference was observed between the combination treatment (p53 *plus* PARP12 knock-down) and PARP12 knock-down alone, suggesting that p53 is dispensable for the observed phenotype.

**Fig. 6:**
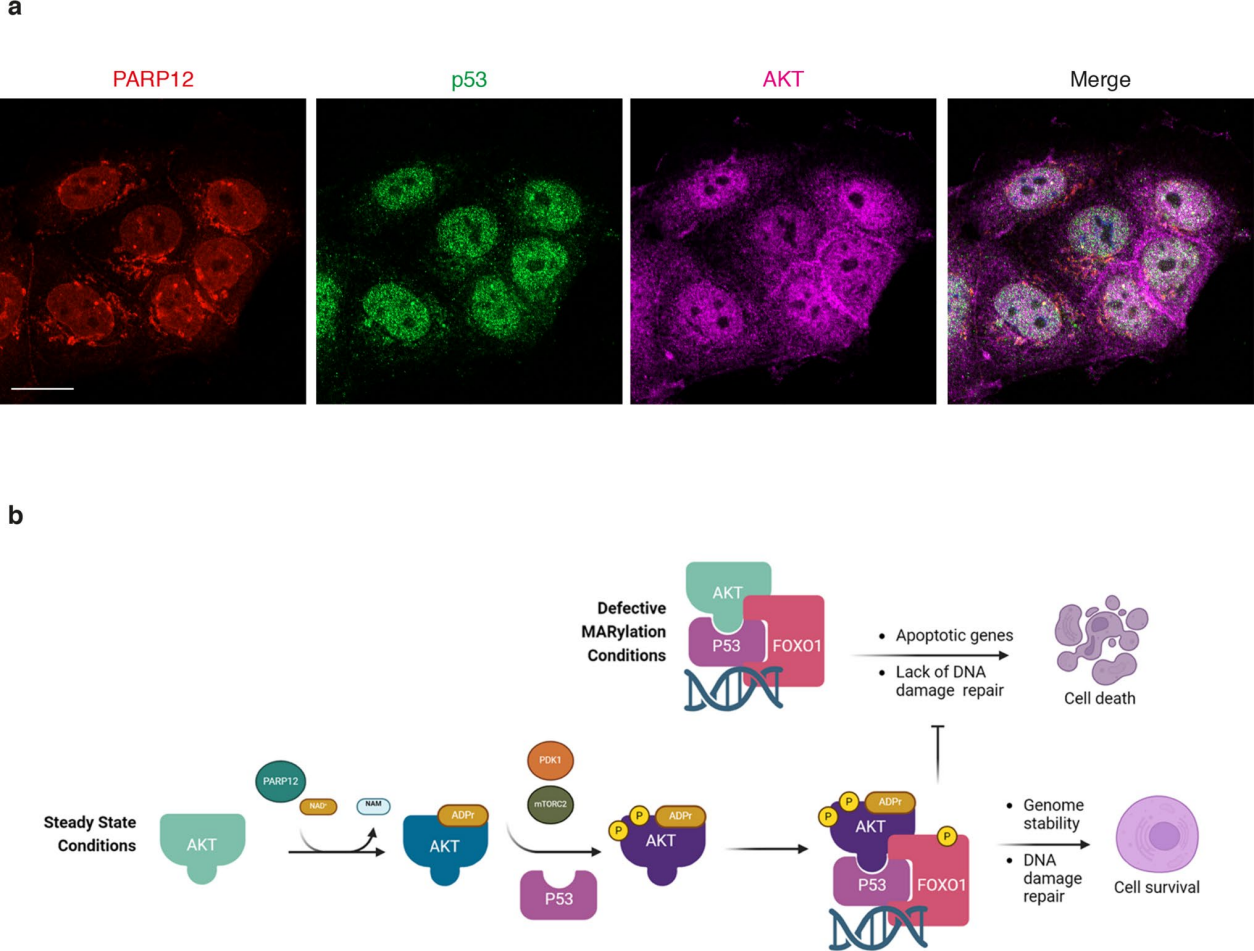
Nuclear localization of AKT, p53 and PARP12. **a** Representative confocal microscopy images of MCF7 cells showing subcellular localization of endogenous PARP12 (red), p53 (green) and AKT (magenta). Scale bar 10 μm. **b** Working model showing the effect of PARP12-mediated ADP-ribosylation on AKT activation and function in cell survival. See text for details

As downstream targets of AKT, we focused on FOXO transcription factors. Active AKT phosphorylates FOXO proteins leading to their nuclear exclusion, degradation and the promotion of cell survival [28, 32]. Therefore, we followed FOXO1 phosphorylation and total levels under PARP12-depletion conditions at different time points (24-48-72 h). As reported in Fig. 5, PARP12 knock-down correlated with reduced FOXO1 phosphorylation levels and increased FOXO1 total levels (Fig. 5 g–i). In turn, as a downstream target of FOXO1, protein levels of Bim were evaluated, that successively showed an increase (Fig. 5 g–j). Interestingly, confocal microscopy analysis reveals the presence of a nuclear pool of PARP12, indicating a potential role for PARP12 in DNA damage-related processes (Fig. 6a). Overall, our data provide evidence for a positive role of PARP12 in regulating AKT activation and promoting cell survival: under unperturbed conditions, PARP12 (hence MARylation) controls AKT activation, required for a functional p53-AKT complex, a prerequisite for genome stability and cell survival. Deprivation of PARP12 causes an imbalance in AKT activation (reduced phosphorylation levels), that reflects in increased FOXO levels and activation of the apoptotic cascade (Fig. 6b). The increased DNA damage can therefore be considered as the consequence of an inefficient AKT, unable to repair basal DNA damage, thereby causing apoptosis.

## Discussion

With this study, we have identified a novel regulation step of AKT activation, mediated by PARP12-catalysed MARylation. Here, AKT is described as a novel substrate of PARP12, a mono-ADP-ribosyltransferase acting as a key factor in mediating breast cancer cell survival [18]. Impairment of AKT MARylation correlates with apoptosis induction of a subset of ER + breast cancer cells, as a consequence of inactive AKT and subsequently increased FOXO nuclear activity. This phenotype is detectable in ER + breast cancer cells, suggesting an interconnection between the oestrogen receptor and PARP12-driven pathways. Seventy percent of all breast cancers exhibit detectable expression of the ER and rely on ER-dependent signalling for proliferation and survival [2]. Hormone-based therapy is therefore the standard of care for ER + breast cancer; however, resistance mechanisms may arise during treatment. Regarding this aspect the observation that PARP12 levels inversely correlate with the survival rate of ER + breast cancer patients already treated with chemotherapy and/or hormone-based therapies is of particular interest, since it does not impinge on the survival rate of ER-negative breast cancer patients. By combining i) our findings showing apoptosis induction in ER + breast cancer cells upon PARP12 knock-down and ii) increased PARP12 gene expression levels observed in patients unable to respond to chemotherapy or to endocrine therapies, we inferred a role of PARP12 in driving resistance, thus proposing this enzyme as a novel potential drug-target to sensitize resistant tumors to current endocrine or combinatorial therapies.

Mechanistically, we demonstrate that PARP12 MARylates AKT, favouring its phosphorylation by its upstream kinases PDK1 and mTORC2. Once activated, AKT phosphorylates downstream targets to carry-out diverse cellular functions that affect cell growth, survival, and metabolism [33–36]. Findings herein reported show that when PARP12 expression is halted, AKT is inactive (due to the reduced phosphorylation levels) leading to increased FOXO protein levels, allowing the activation of the apoptotic cascade. Along a similar line of evidence, the mono-ADP-ribosyltransferase PARP3 has been reported to positively regulate the Rictor/mTORC2 signalling in models of triple negative breast cancer. In detail, PARP3-mediated MARylation of GSK3β restrained its activity to degrade Rictor through ubiquitination, leading to the stabilisation of the mTORC2 complex and enhancing AKT phosphorylation at the serine 473 residue. Despite operating through distinct mechanisms, these findings suggest the existance of a cross-talk between the AKT signalling and PARP-catalysed MARylation, hence corroborating the significance of MARylation in regulating key cellular processes [37].

Using the ADPredict tool [27], we identified two key residues in AKT (E298 and D302) as ADP-ribosylation targets. Biochemical and functional validation of these residues provides robust evidence for a novel regulatory layer involving ADP-ribosylated AKT in the apoptotic phenotype. However, at this stage, we cannot rule out the involvement of additional residues in the modification, which could potentially be identified through refined mass spectrometry analysis [43]. Accurate identification of post-translational modifications is crucial for deciphering signalling pathways. Despite advances in technology, detecting labile forms of ADP-ribosylation, such as ester-linked ADP-ribose, remains challenging [38–44]. More recently, new protocols have enabled the analysis of PARP1-dependent Asp/Glu MARylation during the DNA damage response and suggest a much broader role of this modification in multiple biological pathways [43]. In this context, our findings provide pioneering evidence for MARylation on acidic residues mediated by PARP12, revealing its emerging role in regulating critical cellular pathways [5, this study]. Notably, our data highlight a correlation between DNA damage induction and PARP12 signalling, further underscoring the importance of this modification.

While our findings provide evidence for the involvement of the identified ADP-ribosylated sites in AKT regulation and apoptotic induction, it is important to note that the approach used for identifying these residues overlook other potential ADP-ribosylation target residues, which might be endowed with accessory regulatory functions and hold broader biological relevance. Despite these limitations, our work lays a critical foundation for understanding the role of PARP12 in breast cancer biology.

The apoptotic phenotype induced in the absence of PARP12 can be explained by the stalled AKT-p53 complex due to defective AKT MARylation (*i.e.* halted activation). Whereas a direct interaction between AKT and p53 has been previously reported [31], our data identify ADP-ribosylation (and subsequent phosphorylation) as a novel regulation layer in the complex assembly. Dysregulation events in AKT modification in turn correspond to reduced FOXO1 phosphorylation levels, accounting for activation of the FOXO-dependent apoptotic cascade and impaired repair of DNA damage. We propose PARP12 as part of this regulatory system: its absence counteracts the cytoprotective function of this complex, sensitizing cells to accumulate DNA damage and fostering the apoptotic cascade. Notably, over-expression of the AKT ADP-ribosylation defective mutant M3, as well as the over-expression of the AKT phosphorylation defective mutant (T308A/S473A) or AKT inhibition, phenocopies the effects of PARP12 depletion in MCF7 cells, demonstrating the key role of an inactive AKT in the PARP12-dependent apoptotic phenotype. Of further note, our data also account for an involvement of the oestrogen receptor in this pathway, although its contribution in the described cascade needs to be defined. At present, we cannot rule out a broader involvement of PARP12 in regulating additional components across multiple pathways, which may help explain the molecular determinants responsible for the PARP12-dependent phenotype—an aspect that warrants further investigation. Importantly, our findings support a significant role of PARP12 in DNA damage-related processes. This raises the intriguing possibility that PARP12 knock-down may induce synthetic lethality in cells harboring additional defects in DNA repair mechanisms, potentially explaining the selective sensitivity observed in certain cell subsets.

Our research has uncovered a novel step in the activation of AKT that holds the potential for targeted pharmacological intervention. Activation of the AKT signalling pathway is considered as a cellular defence mechanism employed to counterbalance chemotherapeutic effects [3, 45]. Whilst various PI3K inhibitors have been investigated for treating breast cancer (such as alpelisib, which has gained FDA approval for ER + breast cancer), their efficacy is primarily observed in patients with mutated PI3K and PTEN [46]. On the other hand, they exhibit diminished effectiveness in patients developing resistance mechanisms unrelated to genetic mutations.

In this framework, it is worth mentioning the recent identification of a global activation of the PI3K/ AKT/mTOR signaling axis, specifically upregulated in patients with progressive disease compared to responders treated with endocrine therapy in combination with cyclin-dependent kinase CDK4/6 inhibitor [47]. This study showed a significant increase in the phosphorylation levels of pAKT^T308^- pAKT^S473^- pFOXO1^S246^, proposed here as biomarkers to select patients able to respond to combinatorial therapies [47]. Similar results were obtained in a different clinical trial assessing responses to the AKT inhibitor MK-2206, thus corroborating the significance of phosphoprotein-based biosignatures as predictors of responses to therapies directed towards various components of the PI3K/AKT/mTOR signaling axis [48]. PARP12-mediated regulation of AKT could therefore be of potential interest in patients with progressive disease by representing an alternative regulatory step to be exploited in tumors with short-term/lack of response to current therapies.

In conclusion, our model describes a new regulation step in the PI3K-AKT-FOXO pathway, that selectively occurs in a subset of ER + breast cancer cells. Since the PI3K–AKT–FOXO axis is highly active in the development of breast cancer drug-resistance and PARP12 is a positive regulator of this pathway, we propose PARP12 as potential pharmacological target to be exploited to re-sensitize ER + breast cancer to endocrine treatment.

## Supplementary Information

Below is the link to the electronic supplementary material.

Supplementary file1 (PDF 760 KB)

## Data Availability

The data and materials will be available from the corresponding author upon reasonable request.

## Abbreviations

BIM: Bcl2-interacting mediator
CDK: Cyclin-dependent kinase
ER: Estrogen receptor
FACS: Flourescent-activated cell sorter
FDA: Food and drug administration
FOXO1: Forkhead box O1
GSK3β: Glycogen synthase kinase 3 beta
GST: Glutathione-S-transferase
HA: Hemagglutinin
His: Histidine
MARylation: Mono-ADP-ribosylation
mTORC2: MTOR complex 2
NAD: Nicotinamide adenine dinucleotide
PARylation: Poly-ADP-ribosylation
PDK1: Pyruvate dehydrogenase kinase 1
PI3K: PhosphoInositide-3-kinase
p53: Tumour protein p53
PTEN: Phosphatase and tensin homolog
Rictor: Rapamycin-insensitive companion of mammalian target of rapamycin
