# Supplementary material for: PARP12-mediated ADP-ribosylation contributes to breast cancer cell fate by regulating AKT activation and DNA-damage response
Source: Cell Mol Life Sci. 2025 Jan 23;82(1):58. doi: 10.1007/s00018-025-05586-z (PMC11757654; doi:10.1007/s00018-025-05586-z)
Supplement: Supplementary file 1 — Supplementary file1 (PDF 760 KB) [file 18_2025_5586_MOESM1_ESM.pdf]

Supplementary Figures

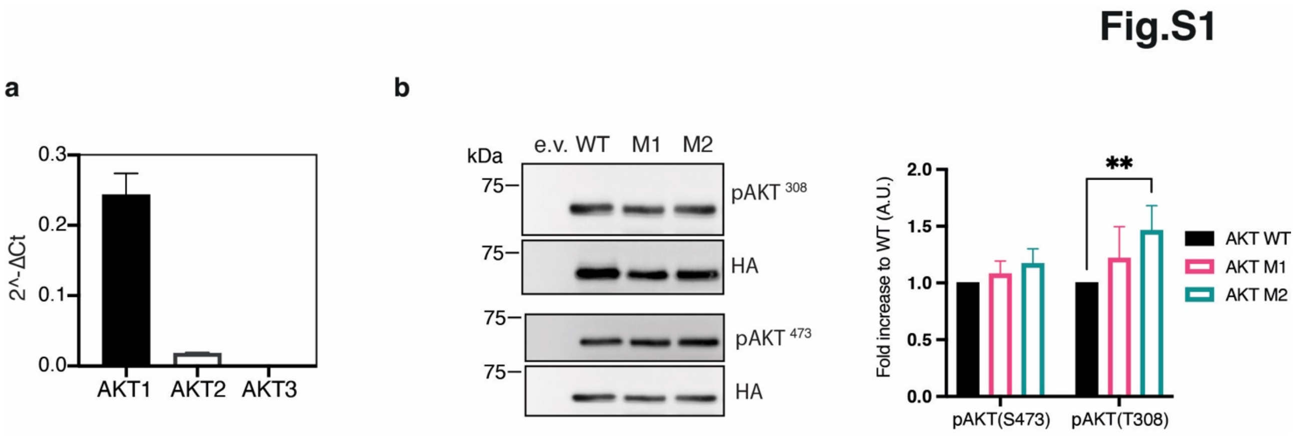

Fig. S1

a. Quantitative real-time PCR analysis (2^-ΔCt values) of AKT1, AKT2 and AKT3 gene expression in MCF7 cells. GAPDH used as housekeeping gene. **b.** Representative western blotting of total lysates from MCF7 cells transfected with WT HA-tagged AKT (WT) or its ADP-ribosylation defective mutant M1 and M2; cells transfected with the empty vector were used as control. Phosphorylated AKT fractions and total AKT are shown (pAKT^{S473}, pAKT^{T308}, HA)**.** The graph shows the quantification of AKT phosphorylation levels. Data are the mean of three independent experiments ± SD. ** P < 0.01 *versus* WT calculated by Student's t test.

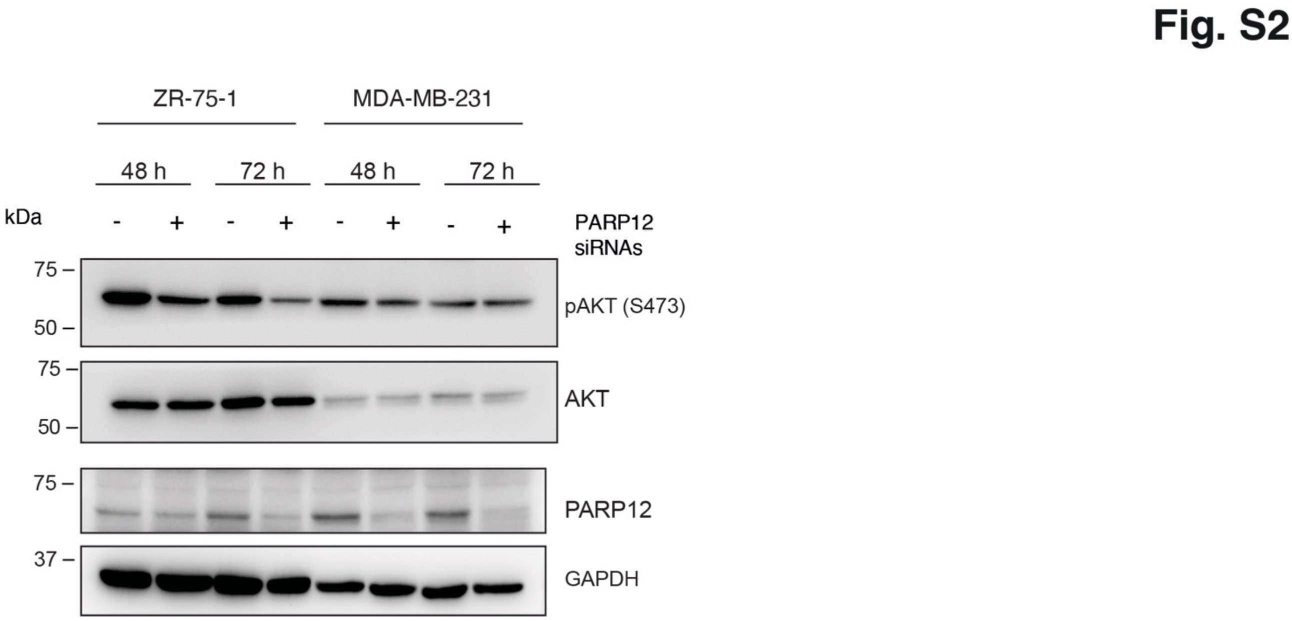

Fig. S2

Representative western blotting analysis of lysates from different cell lines (as indicated) transfected or not with PARP12 siRNAs for 48 and 72 h. PARP1, PARP12, GAPDH, pAKT^{S473} and AKT signals were detected (as indicated). Molecular weight standards (kDa) are indicated on the left of each panel.

Fig. S3

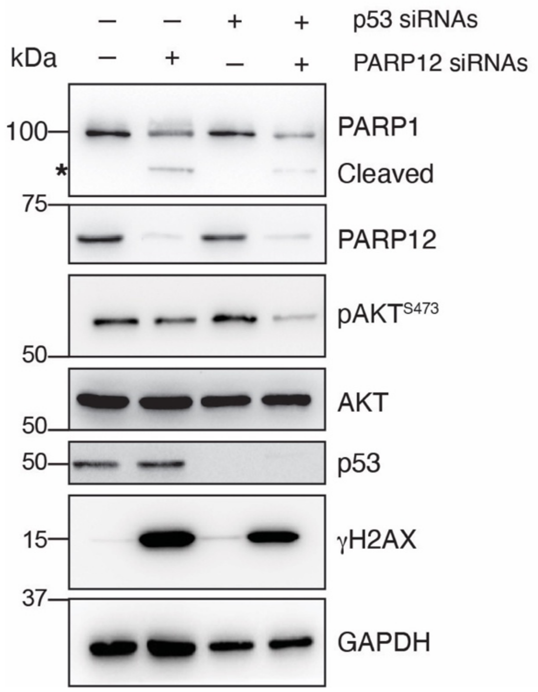

Fig. S3

PARP12 and p53 siRNAs were transfected alone or in combination in MCF7 cells for 72 h. Representative western blotting (antibodies as indicated) of total lysates. Asterisk indicates cleaved PARP1. GAPDH is shown for the internal protein levels; molecular weight standards (kDa) are indicated on the left of each panel. Data are representative of three independent experiments.
